# Fabrication and Characterization of Glucose Biosensors by Using Hydrothermally Grown ZnO Nanorods

**DOI:** 10.1038/s41598-018-32127-5

**Published:** 2018-09-13

**Authors:** Nur Syafinaz Ridhuan, Khairunisak Abdul Razak, Zainovia Lockman

**Affiliations:** 1 School of Materials and Mineral Resources Engineering, Universiti Sains Malaysia, 14300 Nibong Tebal, Penang Malaysia; 2 NanoBiotechnology Research & Innovation (NanoBRI), INFORMM, Universiti Sains Malaysia, 11800 USM Gelugor, Penang Malaysia

## Abstract

Highly oriented ZnO nanorod (NR) arrays were fabricated on a seeded substrate through a hydrothermal route. The prepared ZnO nanorods were used as an amperometric enzyme electrode, in which glucose oxidase (GOx) was immobilised through physical adsorption. The modified electrode was designated as Nafion/GOx/ZnO NRs/ITO. The morphology and structural properties of the fabricated ZnO nanorods were analysed using field-emission scanning electron microscope and X-ray diffractometer. The electrochemical properties of the fabricated biosensor were studied by cyclic voltammetry and amperometry. Electrolyte pH, electrolyte temperature and enzyme concentration used for immobilisation were the examined parameters influencing enzyme activity and biosensor performance. The immobilised enzyme electrode showed good GOx retention activity. The amount of electroactive GOx was 7.82 × 10^−8^ mol/cm^2^, which was relatively higher than previously reported values. The Nafion/GOx/ZnO NRs/ITO electrode also displayed a linear response to glucose ranging from 0.05 mM to 1 mM, with a sensitivity of 48.75 µA/mM and a low Michaelis–Menten constant of 0.34 mM. Thus, the modified electrode can be used as a highly sensitive third-generation glucose biosensor with high resistance against interfering species, such as ascorbic acid, uric acid and L-cysteine. The applicability of the modified electrode was tested using human blood samples. Results were comparable with those obtained using a standard glucometer, indicating the excellent performance of the modified electrode.

## Introduction

Glucose biosensors have extensive industrial, environmental and biomedical applications. In clinical medicine, these biosensors are used to monitor patients’ blood glucose level to diagnose and treat diabetes^1,2^. Diabetes is a metabolic disease that causes an abnormal blood sugar level, which consequently activates several metabolic pathways related to inflammation and apoptosis events^3^. This disease has no cure thus far. Hence, patients with diabetes consistently need to monitor their blood glucose levels to avoid complications. Thus, developing a fast, sensitive and reliable biosensor to detect glucose is necessary. Electrochemical biosensors, especially those containing glucose oxidase (GOx), are the preferred devices for determining blood glucose due to their simplicity, selectivity, sensitivity and direct point care assays^4^. However, challenges in the use of enzyme-based glucose biosensors remain because of the low efficiency of enzyme immobilisation on a solid electrode. To overcome this problem, many researchers have utilised nanomaterials with different structures.

Numerous types of nanomaterials [e.g. nanoparticles (NPs)^5^, nanorods (NRs)^1,6^, nanotubes (NTs)^7^, nanowires^8^ and nanosheets^9^] are used as glucose biosensors because of their high surface-area-to-volume ratios, which enable high loading and provide a responsive microenvironment for stabilising and preventing the leakage of immobilised enzymes^10^. Sensitivity is also enhanced due to the efficient electron transfer from the enzyme to the electrode and the ability to include an additional catalytic step for fast electron transfer^3^. According to Sun *et al*., 1D nanostructures, such as NTs, nanowires and nanorods can provide more natural channels that benefit electron transfer than NPs and nanofilms^11^. 1D metal oxide nanostructured materials have gained increasing interest because they provide a biocompatible electroactive surface for efficient enzyme immobilisation and their strong absorption ability^12^. Metal oxide nanomaterials possess high surface area and isoelectric point (IEP), leading to a good and reliable surface for immobilisation of GOx with low IEP (~4.2). Several high-IEP metal oxide nanomaterials used in glucose detection are ZnO^13,14^, TiO_2_^15–17^, CuO^18–20^, CeO_2_^21,22^ and ZrO_2_^23,24^.

ZnO is a promising electrode material used to fabricate electrochemical glucose biosensors because of its biocompatibility and excellent properties, such as low toxicity, high electron mobility and easy fabrication^2,25^. ZnO also possesses an IEP of ~9.5 which is suitable for adsorption of low-IEP enzymes, particularly GOx (IEP: ~4.2–4.5), at the physiological pH of 7.4 through electrostatic attraction^2,4,25^. Enzyme redox capability is always hindered when the redox centre is insulated. Therefore, electron transfer does not occur directly if no redox mediator is used. However, the use of ZnO provides direct electron transfer (DET) without using a redox mediator because the electrode and enzyme operate in a small potential window closer to the redox potential of the enzyme itself, thereby causing the biosensor to be less prone to other interfering biomolecules^26^. Many studies on glucose sensors involve different ZnO nanostructures with different morphologies, such as NPs^5,27,28^, nanofilms^29^, nanosheets^9^, nanocombs^30^ and NTs^7^. These nanostructures offer a good surface for enzyme immobilisation but show relatively poor stability because they can be easily removed from the substrate during functionalisation^31^. Adherence can be improved by growing nanorods directly on substrates rather than transferring loose nanorods onto substrates. Therefore, nanorod structure is ideal for enzyme immobilisation because it enables direct and fast electron transport between the electrode substrate and enzyme^32^. Nanorods grown directly on a substrate also provide good stability because the process is chemically and mechanically robust^33^.

Numerous methods can be used to grow ZnO nanorods, but the hydrothermal method is the most advantageous because it is simple, requires low temperature and has low cost. This hydrothermal method is also surface-independent and thus can provide good control the morphology of the grown nanorods^34^.

The use of ZnO nanorods grown through hydrothermal method for glucose biosensors has been reported^1,2,13,14,35–38^. Chou *et al*.^1^ and Ma *et al*.^14^ used electron mediators to enhance glucose detection performance. Jung *et al*.^37^ used a cross-linker on GOx immobilisation. Lei *et al*.^2^ used physical adsorption for GOx immobilisation. However, ZnO nanorods are indirectly grown on a substrate, thereby affecting the electron-shuttling path between the enzyme and substrate. Most previous works used an electron mediator and additional steps in GOx immobilisation to increase glucose detection performance.

In our previous work^39^, we reported our preliminary finding on the influence of ZnO NRs with a high surface area for glucose detection without optimising the fabrication parameters and information on electrochemical and electrocatalytic properties. In the present paper, we reported thorough analyses of glucose detection by using amperometric technique, analyses of interference, reusability and reproducibility performance and quantification of real samples (blood sample). Such analyses have not been reported before. The mechanism of glucose detection and scientific explanation were also presented.

In this work, ZnO nanorods were grown directly on a seeded substrate through hydrothermal method and were used as a modified electrode for glucose detection. Seeded substrates were prepared by sol–gel method, wherein a seed layer was coated onto ITO substrates. This method is simple, economic and effective and suitable for producing a high-quality ZnO seed layer for growing nanostructures by hydrothermal method. The ITO glass substrate was used because it is cheaper than Au or glassy carbon electrodes. ITO glass also has low resistivity and can be easily shaped and trimmed to facilitate fabrication^40^. Physical adsorption was used to immobilise GOx onto nanorods by exploiting the high IEP of ZnO (pI of ~9.5) suitable for low GOx enzyme with low IEP (pI: ~4.2–4.5). Our method does not use redox mediators, thereby ensuring electrode stability. Redox electron mediators enable electron transfer from GOx towards electrode, but they also decrease the stability of the electrode. To our knowledge, previous works did not present comparative studies on parameters influencing enzymatic activity and biosensor performance (e.g. temperature, pH and enzyme concentration). The modified electrode showed good performance and stability in detecting glucose through electrocatalytic response. Even after 14 days of use, the modified electrode retained 85% of its original performance, which is comparable with the results of Zhao *et al*.^41^ and Zhou *et al*.^42^ (i.e. 87% and 80.8%, respectively). The modified electrode also showed good reliability towards glucose detection in real blood samples.

## Results and Discussion

### Characterization and morphology of ZnO nanorod matrix

**Figure 1:**
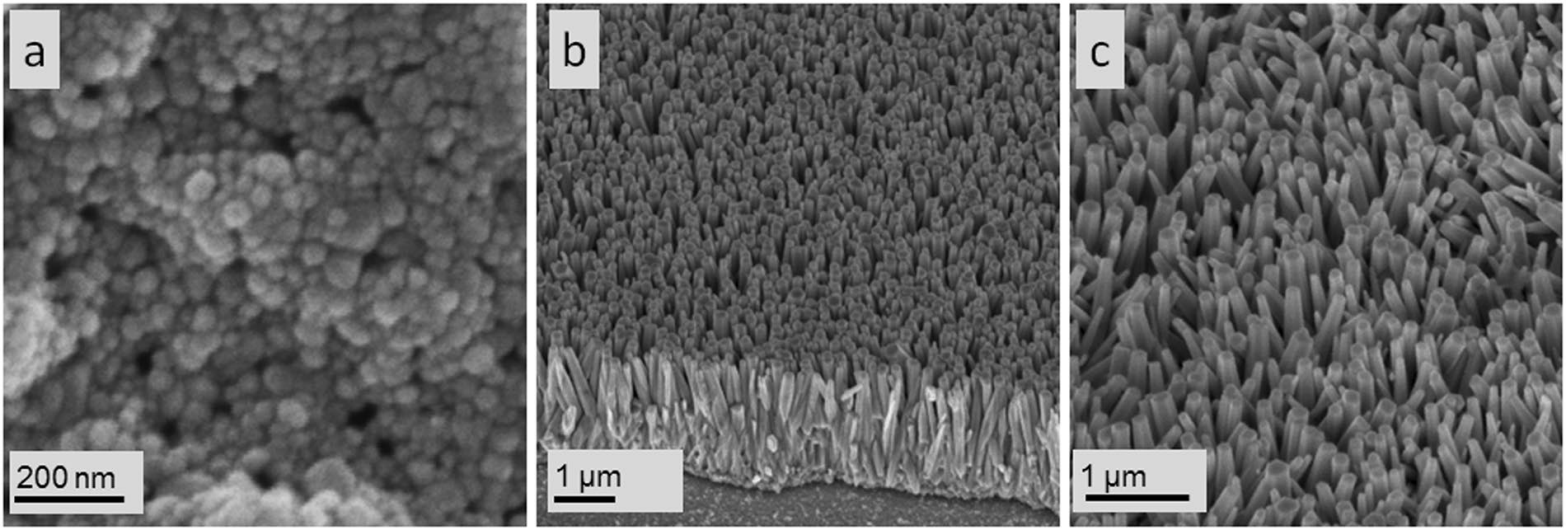
FESEM images of (**a**) ZnO seed layer, and (**b**,**c**) top and cross-section, respectively, of ZnO nanorod growth through hydrothermal process.

ZnO nanorods were successfully grown on a ZnO seed layer (Fig. 1). Figure 1(a) shows the field-emission scanning electron microscopy (FESEM) image of the surface morphology of the ZnO seed layer deposited onto the ITO glass. The prepared ZnO seed layer consisted of homogenous and spherical NPs that were uniform in size and had an average diameter of 85 nm. The seeds were also not too compact and provided sufficient space for the growth of ZnO nanorods during the hydrothermal reaction. Seed layer is crucial to the hydrothermal process because it is involved in achieving uniform distribution and growth orientation of the nanorod structure. Polsongkram *et al*.^43^ reported that the absence of seed layer during hydrothermal growth results in the random growth of ZnO nanorods, with most of them assembling into branch-like morphology. Although nanorods can be formed, the size distribution and dimensions of the nanorods can be relatively poor. This phenomenon is due to the lattice mismatch and high surface energy for ZnO nanocrystals to be deposited onto a bare substrate. Accordingly, in the present work, ZnO seed layers were used to control the morphology and orientation of the grown nanorods. The uniform surface roughness of ZnO seed layer ensured homogeneity and high density of grown nanorod array [Fig. 1(b) and (c)]. Compared with the seed layer morphology, the surface oxide possessed rod-like structure after 4 h of hydrothermal reaction and average diameter and length of 109.9 and 645.4 nm, respectively. The FESEM image of ZnO nanorods viewed at 90° revealed that the growth of nanorods was perpendicular to the ITO substrate.

In this work, Zn(NO_3_)_2_ and hexamethylamine (HMT) were used as the primary source of Zn^2+^ and OH^−^ ions, respectively. During hydrothermal reaction, Zn^2+^ ions were supplied through the dissolution of Zn(NO_3_)_2_ (Equation 1). HMT thermally decomposed into HCHO and NH_3_ which was then hydrolysed into NH_4_OH to supply OH^−^ ions, as shown in Equations 2 and 3. When the amount of OH^−^ ions was sufficient, and the hydrothermal reaction reached supersaturation, Zn^2+^ ions started to react with OH^−^ ions to form several monomeric hydroxyl species, including ZnOH^+^ (aq), Zn(OH)_2_ (aq), Zn(OH)_2_ (s), Zn(OH)^−^_3_ (aq) and Zn(OH)^2−^_4_ (Equation 4 and 5). The chemical reactions that occurred are illustrated by the following equations^44,45^:

1 $${\rm{Zn}}{({{\rm{NO}}}_{{\rm{3}}})}_{{\rm{2}}}\cdot {{\rm{6H}}}_{{\rm{2}}}{\rm{O}}\to {{\rm{Zn}}}^{2+}+{\rm{2}}{({{\rm{NO}}}_{{\rm{3}}})}^{-}+{{\rm{6H}}}_{{\rm{2}}}{\rm{O}}$$

2 $${{\rm{C}}}_{{\rm{6}}}{{\rm{H}}}_{{\rm{12}}}{{\rm{N}}}_{{\rm{4}}}+{{\rm{6H}}}_{{\rm{2}}}{\rm{O}}\leftrightarrow {\rm{6CHCHO}}+{{\rm{4NH}}}_{{\rm{3}}}$$

3 $${{\rm{NH}}}_{{\rm{3}}}+{{\rm{H}}}_{{\rm{2}}}{\rm{O}}\leftrightarrow {{{\rm{NH}}}_{{\rm{4}}}}^{+}+{{\rm{OH}}}^{-}$$

4 $${{\rm{Zn}}}^{2+}+{\rm{4}}({{\rm{OH}}}^{-})\to {\rm{Zn}}{{({\rm{OH}})}^{{\rm{2}}-}}_{{\rm{4}}}\to {\rm{ZnO}}({\rm{s}})+{{\rm{H}}}_{{\rm{2}}}{\rm{O}}+{\rm{2}}({{\rm{OH}}}^{-})$$

5 $${{\rm{Zn}}}^{2+}+{\rm{2}}({{\rm{OH}}}^{-})\to {\rm{Zn}}{({\rm{OH}})}_{{\rm{2}}}\to {\rm{ZnO}}({\rm{s}})+{{\rm{H}}}_{{\rm{2}}}{\rm{O}}$$

The formation of ZnO nanorods by hydrothermal process involved two stages, namely, nucleation and growth. During nucleation, ZnO(s) nuclei were produced by the dehydration of several monomeric hydroxyl ions as mentioned before. Then, these ZnO(s) were deposited onto the existing seed layer through heterogeneous crystallisation which then initiated nanorod growth. After nucleation, nanorod growth continued through the condensation of zinc hydroxyl complexes, including Zn(OH)^2−^_4_ and Zn(OH)_2_^45^. The nanorods grew into flat-tip hexagonal structures. ZnO possesses polar hexagonal wurtzite structures comprising a positively charged polar surface of (0001) and nonpolar surface of six-sided planes [Fig. 1(b)]. During growth, the negatively charged Zn(OH)^2−^_4_ ions were strongly attracted to the positively charged (0001) surface. Subsequently, growth occurred on the c-axis direction upon the condensation of Zn(OH)^2−^_4_ to the ZnO nanocrystals on the (0001) plane. At the same time, HMT acted as a chelating agent adsorbed onto the nonpolar surface, thereby hindering the radial enlargement of rods and inducing growth vertically, that is along the c-axis. This phenomenon resulted in the formation of an aligned nanorod array [Fig. 1(c)].

**Figure 2:**
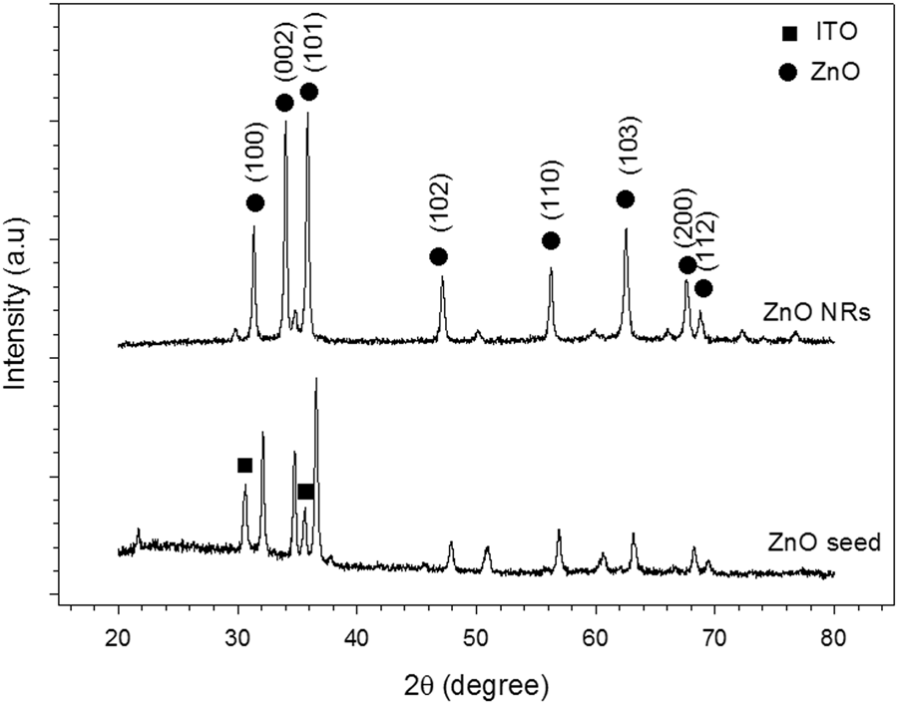
XRD patterns of ZnO seed layer and synthesised ZnO nanorods via hydrothermal process.

The X-ray diffraction (XRD) patterns of the ZnO seed layer and the grown nanorods are illustrated in Fig. 2. The XRD peaks attributed to ZnO were detected at 2θ values of 31.7°, 34.4° and 36.2°, corresponding to the (100), (002) and (101) planes, respectively, for both samples. This pattern was consistent with wurtzite crystal structure (ICDD 36-1451). The ZnO seed layer pattern had higher intensity of the (101) plane than that of the (002) plane. This finding was due to the random atomic arrangement of glass substrate that may have disturbed the orientation of ZnO seed layer, resulting in a small incline in the orientation of the seed layer. This result agreed with that of Chakrabarti *et al*.^46^, indicating that the substrate used can influence the preferred orientation in sol–gel-derived ZnO films due to the glass substrate with nonbridging oxygen. The intensity of the (002) plane in ZnO nanorods increased after hydrothermal reaction compared with ZnO seed layer because of the preferred growth in the c-axis direction. However, the intensity of the (101) plane was slightly similar to that of the (002) plane due to initial nuclei alignment. When the (101) plane of ZnO nanorods was initially grown on the (002) axis of ZnO seed layer, ZnO nanorods grew in the c-axis and along the length but slightly inclined. This result agreed with the FESEM images indicating the growth of ZnO nanorods with inclined alignment [Fig. 1(b and c)].

### Electrochemical characterisation of the fabricated glucose sensor

**Figure 3:**
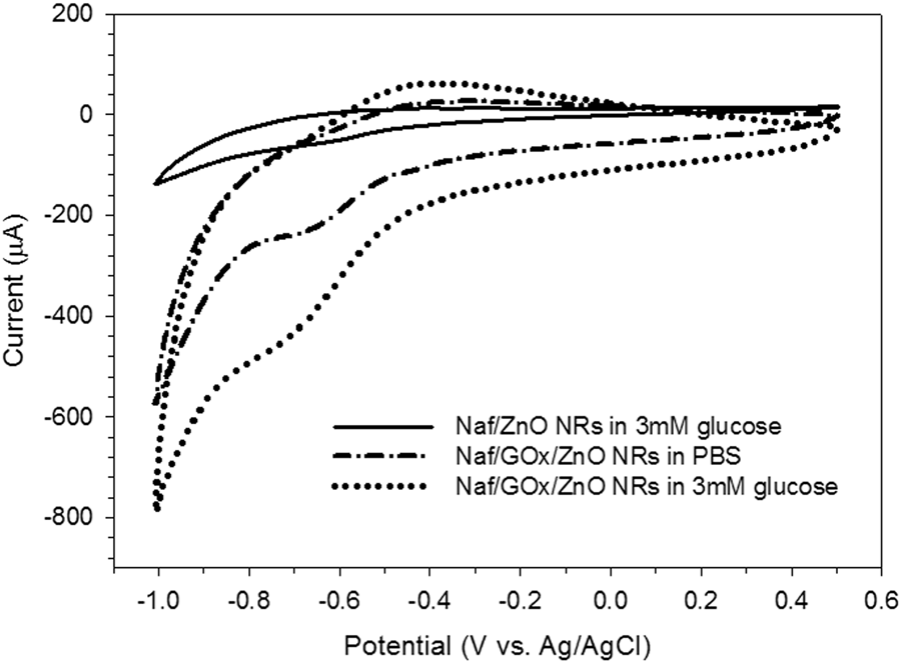
Cyclic voltammograms of Nafion/ZnO NR-modified electrode in 3 mM glucose (solid line) and Nafion/GOx/ZnO NR-modified electrode in 0.01 M PBS (absence of glucose) and 3 mM glucose at scan rate of 50 mV/s.

Cyclic voltammetry (CV) was used to measure the response of electrode in the presence and absence of 3 mM glucose in 0.01 M phosphate-buffered saline (PBS) solution to evaluate the sensing performance of the fabricated electrode with and without GOx immobilisation. Figure 3 shows the CV of Nafion/ZnO NR-modified electrode in 3 mM glucose solution (solid line) at a scan rate of 50 mV/s. In the absence of GOx, a relatively minor anodic current peak at a potential of −0.7 V was observed. In the absence of GOx enzyme on ZnO nanorod surface, the atoms on the nanorod surface acted as the electrocatalyst. Firstly, the available O in the glucose environment was adsorbed onto the nanorod surface, and the ZnO nanorod surface was covered by monolayer O (Equation 6). The adsorbed O was ionised and converted into dynamic O species (O^−^/O^−^_2_) by extracting the ions from the ZnO surface, as shown in Equation 7. Simultaneously, glucose was catalysed by releasing O^−^ to glucono-δ-lactone. The possible mechanism was proposed by Dar *et al*.^47^, as follows:

6 $${{\rm{O}}}_{{\rm{2}}}\leftrightarrow {{\rm{O}}}_{{\rm{2}}}\,{\rm{ads}}\,({\rm{ZnO}})$$

7 $${{\rm{O}}}_{{\rm{2}}}\,{\rm{ads}}\,({\rm{ZnO}})+{{\rm{2e}}}^{-}({\rm{ZnO}})\leftrightarrow {{\rm{2O}}}^{-}\,{\rm{ads}}\,({{\rm{O}}}^{-}/{{{\rm{O}}}^{-}}_{{\rm{2}}})$$

8 $${\rm{Glucose}}+{{\rm{O}}}^{-}\to {\rm{Glucono}} \mbox{-} {\rm{\delta }} \mbox{-} {\rm{lactone}}+{{\rm{2e}}}^{-}$$

The prepared electrode can be utilised for glucose detection even without GOx. However, the applied potential for glucose to catalyse was higher without GOx than with GOx. This phenomenon may have caused selectivity- and sensitivity-related problems later on because other interfering species, such as ascorbic acid (AA) and uric acid (UA), can also catalyse at high potential. GOx was used to ensure the specificity and good performance of the fabricated electrode for glucose detection. As shown in Fig. 3, anodic current increased with a pair of well-defined and quasi-reversible redox peaks observed in the presence of immobilised GOx on ZnO nanorod surface (dotted line). This result can be attributed to the increase in oxidation of glucose which produced free electrons and rapidly transported the produced electron ZnO which was tunnelled towards the substrate to generate current during electrochemical reaction. Low peak potential value was beneficial because it can prevent species from interfering with glucose detection.

To evaluate the performance of GOx immobilisation onto ZnO nanorod surface, we conducted CV comparison in the absence of 3 mM glucose (dotted-dash line) and presence of 3 mM glucose (dotted line). Upon the addition of 3 mM glucose, the shape of CV changed with increased oxidation peak current compared with a peak in the absence of glucose. This result indicated that immobilised GOx was sensitive to the glucose environment. The biocatalytic reaction of immobilised GOx onto the ZnO nanorod surface began with GOx catalysing the oxidation of glucose by causing a reduction in the flavin adenine dinucleotide (FAD) to FADH_2_ that was present in GOx (Equation 9). This process was followed by the reoxidation of reduced flavin with dissolved oxygen to produce flavin oxidase and release 2e^−^, as shown in Equation 10^33^:

9 $${\rm{Glucose}}+{\rm{GOx}}\,({\rm{FAD}})\to \mathrm{Glucono} \mbox{-} {\rm{\delta }} \mbox{-} \mathrm{lactone}+{\rm{GOx}}\,({{\rm{FADH}}}_{{\rm{2}}})$$

10 $${\rm{GOx}}\,({{\rm{FADH}}}_{{\rm{2}}})\to {\rm{GOx}}\,({\rm{FAD}})+{{\rm{2H}}}^{+}+{{\rm{2e}}}^{-}$$

The fabricated Nafion/GOx/ZnO NRs/ITO electrode followed a third-generation glucose sensor mechanism. The high surface area of nanorod structures entrapped and encompassed GOx enzymes, thereby contributing to DET between the GOx enzymes to the modified electrode. Considering the short distance between the morphological structure of nanorods and the active reaction site of enzyme, electrons tunnelled through ZnO even at low operating potential through DET. This observation agreed with that obtained by Nirmal and Swapan^32^. The fabricated electrode showed a high current in glucose detection because of the ZnO nanorod array which possessed high-aspect-ratio surface for GOx to immobilise onto and good alignment of ZnO nanorods for electron to transfer onto the substrate.

### Optimising the parameters for biosensor performance

**Figure 4:**
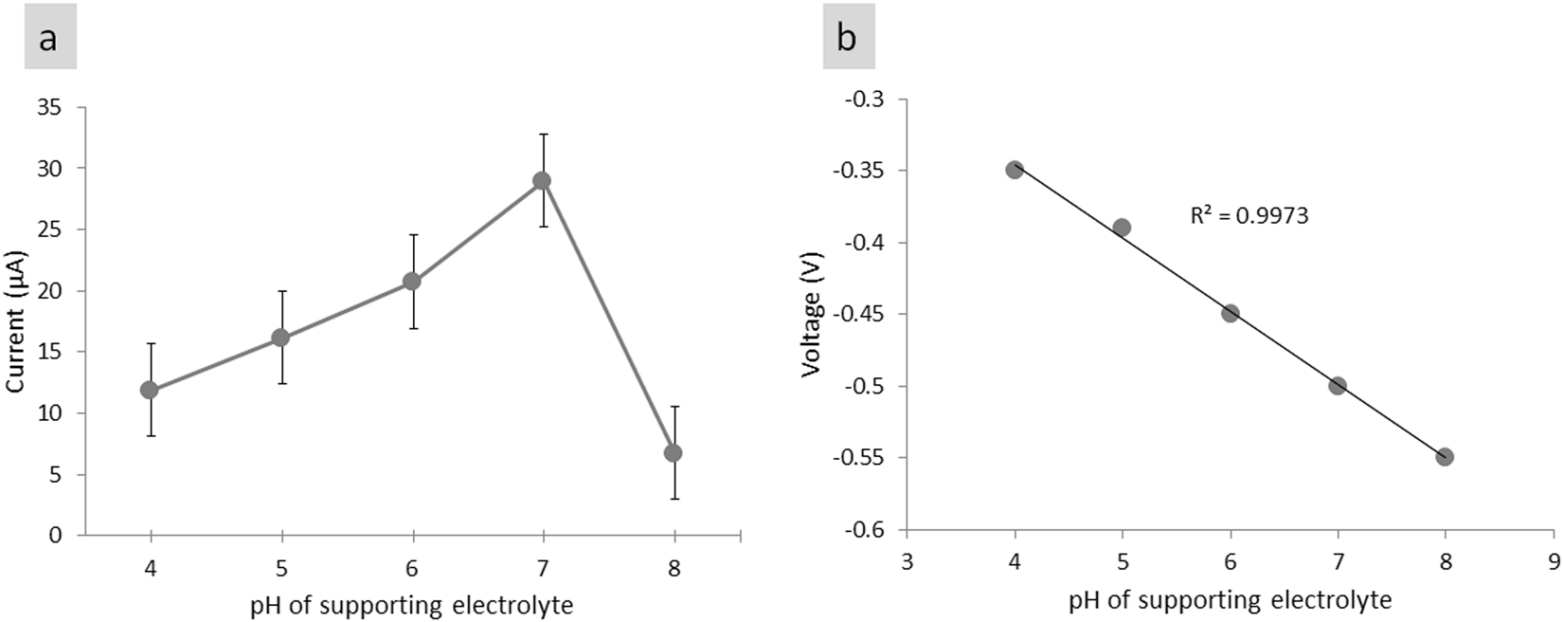
Effect of supporting electrolyte pH from pH 4 to 8 on Nafion/GOx/ZnO NR-modified electrode response to 3 mM glucose. (**a**) The corresponding calibration plot of peak current vs. supporting electrolyte pH, and (**b**) The corresponding calibration plot of potential vs. supporting electrolyte pH.

To fabricate an efficient biosensor for glucose, we studied the supporting electrolyte’s pH solution, temperature and enzyme concentration on the response of Nafion/GOx/ZnO NRs/ITO. The influence of supporting electrolyte pH solution on the redox behaviour of GOx immobilised onto ZnO NR-modified electrode was carried out by CV in 0.01 M PBS solution with different pH values containing 3 mM glucose at a scan rate of 50 mV/s, as shown in Fig. 4(a). A total of 1 M HCl and 1 M NaOH were used to adjust the pH of PBS before adding 3 mM glucose solution. Enzymatic activity is generally pH-dependent because the enzyme is the most stable between the pH values of 3.5 and 8.0 and loses its activity at a pH of more than 8 and less than 2^48^. As shown in Fig. 4, the electrocatalytic activity increased from pH of 4 to a pH of 8 and peaked at approximately pH of 7. According to Bright and Appleby^49^, the optimum pH of free GOx is 5.5; however, pH may shift to 7 due to the substantial difference in the ionic environment of the matrix around the enzyme active site^50^. In the present work, the catalytic activity of GOx decreased at pH > 7 because of the irreversible denaturation of the enzyme, as observed in previous studies^4,13,48^. Fig. 4(b) shows the formal potential of the electrode and the solution pH with a calculated slope value of −52.7 mV/pH and a linear relationship (R^2^ = 0.998). The obtained slope value was close to the theoretical value (−58.6 mV/pH) of the Nernstian equation^51^. The DET of the redox couple at GOx involved an equal number of protons (H^+^) and electrons (e^−^) transferred to the electrode surface which similar to the result observed by Velmurugan *et al*.^51^. The supporting electrolyte’s optimum pH of 7 was selected as the optimum pH for subsequent parameter optimisation.

**Figure 5:**
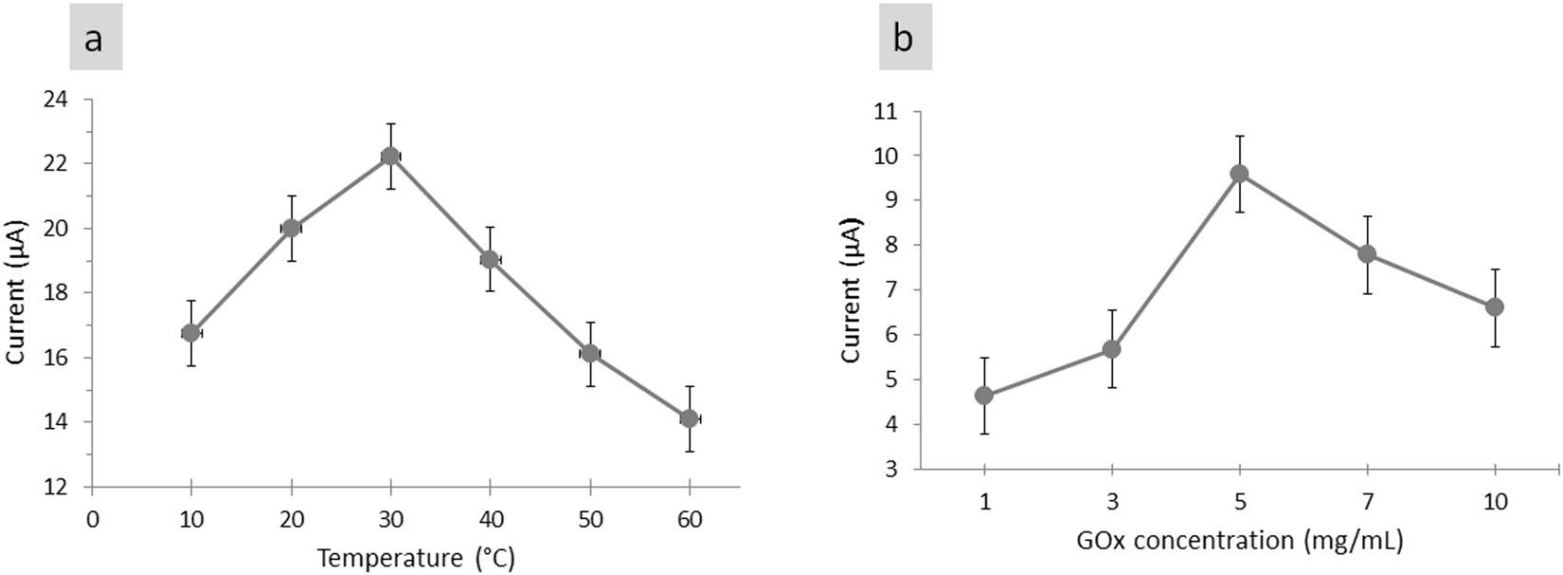
Effect of (**a**) glucose solution temperature from 10 °C to 60 °C and (**b**) GOx enzyme concentration within 1–10 mg/mL used for immobilization on Nafion/GOx/ZnO NRs response in 3 mM glucose solution at scan rate of 50 mV/s.

Enzymes are sensitive to changes in temperature because the reaction rates of enzyme and temperature are exponential^52^. The effect of temperature on sensor performance was also studied by varying the temperature from 10 °C to 60 °C [Fig. 5(a)]. The current response of our sensor gradually increased with increased temperature and peaked at ~30 °C. A drastic drop in current response was observed from 40 °C to 60 °C. The increment of current response with increased temperature is due to the enhanced enzymatic activity and a decrease in dissolved O_2_ concentration^4^. With increasing temperature, the rate of chemical reaction in enzyme also increased as increased amount of energy was gained for enzyme to catalyse. The rate of enzyme catalysed increases thrice every 10 °C increase in temperature^53^. The kinetic energy of GOx enzymes increased with increased temperature, thereby causing an increased amount of glucose to be catalysed and producing an increased number of free electrons. Consequently, the current response increased. Beyond 30 °C, the current response decreased because of the heating effect of the immobilised enzyme that degraded the enzyme’s functionality. This finding agreed with that of Ang *et al*.^48^. At high temperatures, the enzyme can denature and lose its activity because the internal energy of GOx molecule increased as the temperature of the system increased. The internal energy of enzyme molecules may include the translational, vibrational and rotational energies of molecules and the energy involved in the chemical bonding of molecules. As temperature increased, the vibrational and rotational energies of molecules also increased, thereby straining weak bonds, such as H and ionic bonds and causing them to easily break. Breaking bonds within enzyme induced a change in the shape of active sites in GOx and further inactivated the enzyme. The change in shape indicated that the active site of GOx was poorly complementary to the shape of glucose, causing a weak reaction to occur and further decreasing the product yield. Most enzymes were denatured and lost their activity at 40 °C to 70 °C. For the GOx from *Aspergillus niger* (the enzyme used in this study), the optimum temperature ranged from 40 °C to 60 °C^52^. Therefore, a constant temperature of 30 °C was chosen for all subsequent analyses.

To study the effect of the amount of GOx enzyme used for immobilisation, we varied this amount from 1 mg/mL to 10 mg/mL in 3 mM glucose [Fig. 5(b)]. Increasing the enzyme concentration increased the reaction rate as an increased number of GOx enzymes catalysed the glucose molecule. However, this glucose concentration had an effect only up to a certain concentration depending on the glucose molecule concentration. As shown in Fig. 5(b), the current response increased with increased GOx concentration and peaked at 5 mg/mL. The result was due to the kinetically limited reaction of GOx, wherein oxygen and glucose consumption were directly proportional to GOx concentration^54^. With increased GOx concentration, an increased number of free glucose was catalysed, resulting in increased current signal. Further increase in GOx concentration did not change the current response as the reaction was limited by diffusion because not all enzyme molecules can participate in the glucose-catalysed reaction^54^. The enzymes that did not participate in glucose oxidation caused a local decrease in pH, resulting in buffering capacity (insulating barrier) to occur. This buffering capacity reduced the current response produced. This finding agreed with that of Ang *et al*.^48^, who stated that high concentrations of immobilised enzyme results in support overloading.

**Figure 6:**
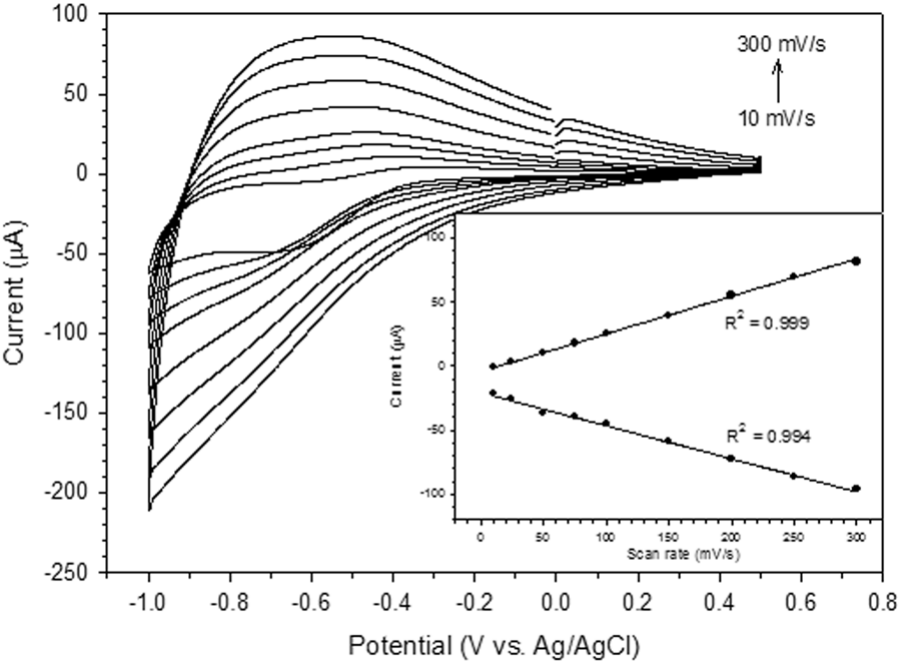
Cyclic voltammograms of Nafion/GOx/ZnO NR-modified electrode in PBS solution (0.01 M, pH 7) at different scan rates within 10–300 mV/s, Inset: corresponding current plots of anodic and cathodic peaks as a function of scan rate.

Figure 6 shows the effect of various scan rates on the electrochemical response of GOx enzyme on the modified Nafion/GOx/ZnO NRs/ITO electrode. The anodic (oxidation) peak potentials shifted to positive potentials, whilst the cathodic (reduction) peak potentials shifted to negative potentials with increasing scan rate, suggesting that a quasi-reversible process occurred at the modified electrode. The ratio of the anodic to cathodic peak current was 0.84. Hence, the electrochemical oxidation of glucose at the modified electrode (Nafion/GOx/ZnO NRs/ITO electrode) is a quasireversible process in which both oxidation (I_pa_) and reduction (I_pc_) peak currents increased as the scan rate increased^55^. The plotted relationship demonstrated that current response was linearly proportional to the scan rate ranging within 10–300 mV/s (Fig. 6, inset). The linear regression equations were I_pa_ (µA) = 0.294ν (mV/s) − 4.307 (R^2^ = 0.999) and I_pc_ (µA) = −0.257ν (mV/s) − 20.833 (R^2^ = 0.994), where ν is the scan rate. This finding indicated that the electron transfer process for the modified electrode was a surface-confined mechanism. The electrons were able to transfer easily between GOx enzyme and ZnO nanorod matrix. This result also proved that GOx enzyme was successfully immobilised onto the ZnO nanorod surface and retained its bioactivity. The average surface concentration (Γ) of the electroactive site of GOx enzymes on the ZnO nanorod matrix surface can be estimated based on the slope of I_p_ versus v (Brown–Anson model)^56^.

11 $${I}_{p}=\frac{{n}^{2}{F}^{2}vA{\rm{\Gamma }}}{4RT}$$

where n is the number of electrons transferred, F is the Faraday constant (96485.34 C/mol), A is the surface area of electrode (1 cm^2^), R is the gas constant (8.314 J/mol·K), T is the absolute temperature (298 K) and I_p_/v is the slope of the calibration plot (scan rate value). The Γ of Nafion/GOx/ZnO NRs/ITO electrode was 7.82 × 10^−8^ mol/cm^2^ which was higher than those obtained using GOx immobilised onto Ag-doped ZnO nanorods (2.42 × 10^−11^ mol/cm^2^) by other researchers^57^ and double-layer GOx immobilised onto ZnO NPs and MWCNTs (6.49 × 10^−10^ mol/cm^2^)^5^. High average surface concentration was due to the high surface area of ZnO nanorods that ensured high loading of GOx immobilisation.

### Amperometric detection of the Naf/GOx/ZnO NRs/ITO electrode glucose

**Figure 7:**
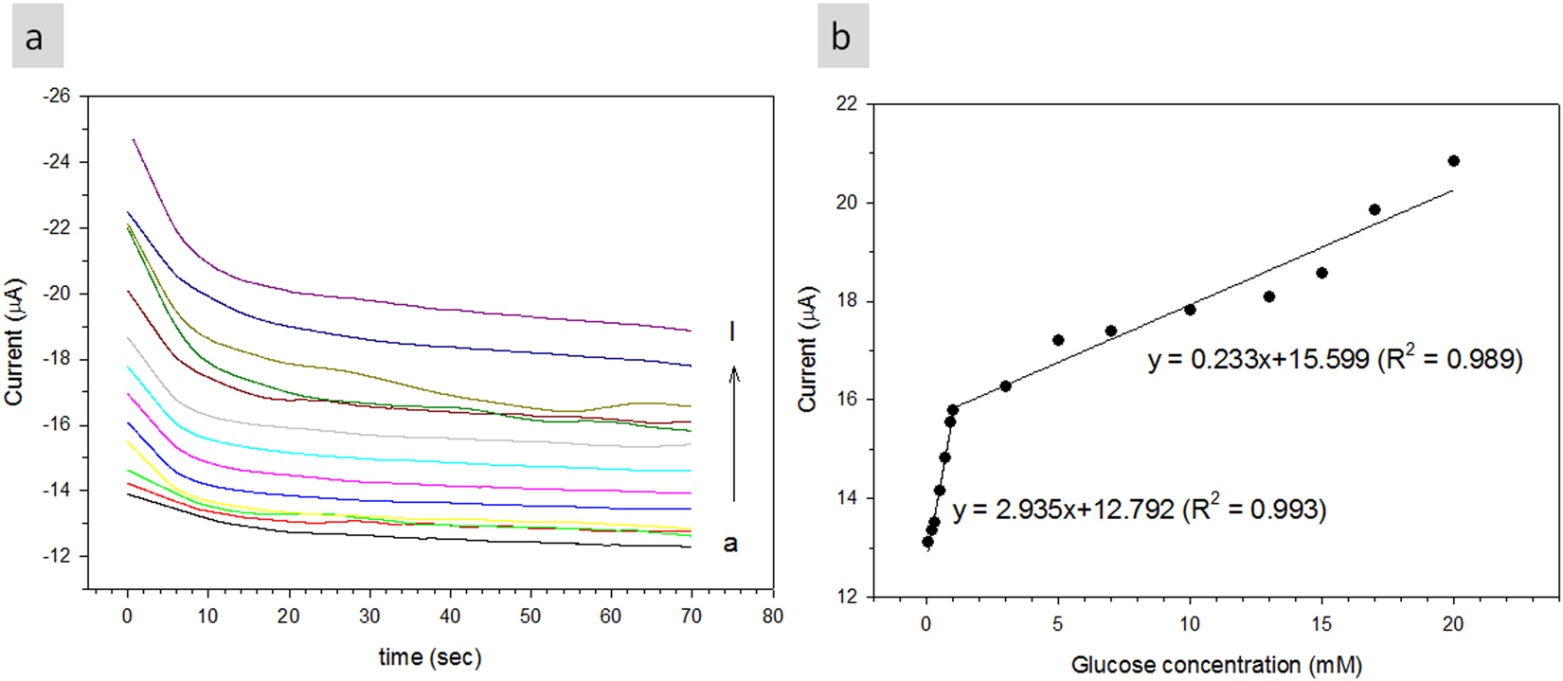
(**a**) Amperometric response of Nafion/GOx/ZnO NR-modified electrode in the presence of different glucose concentrations (from a to i denote as 0.05 to 20 mM), and (**b**) calibration curve of peak current vs. glucose concentration.

Figure 7(a) shows the amperometric response of modified electrode (Nafion/GOx/ZnO NRs/ITO) on the successive addition of glucose (from 0.05 mM to 20 mM) into continuously stirred 0.01 M PBS solution at an applied potential of −0.5 V. Nafion/GOx/ZnO NRs/ITO electrode achieved consistent current response after 10 s which indicated good ability of the modified electrode for electrocatalytic oxidative and swift electron exchange. The current response of the glucose biosensor also increased with increased glucose concentration. Figure 7(b) shows the calibration plot of response current to different glucose concentrations of Nafion/GOx/ZnO NRs/ITO electrode. A good linear response with increased glucose concentration was obtained within 0.05–1.00 and 1–20 mM with sensitivities of 48.75 and 3.87 µA/mM·cm^2^, respectively, with a calculated limit of detection of 0.06 mM on the basis of the following equation^58^:

12 $$LOD=\frac{3.3\sigma }{S}$$

where σ is the standard deviation of response at low concentration, and S is the slope of the calibration curve. To evaluate the biological activity of immobilised GOx, we used the apparent Michaelis–Menten constant ${{\rm{K}}}_{{\rm{M}}}^{{\rm{app}}}$ and calculated it according to the Lineweaver–Burk equation, as follows^14,38^:

13 $$\frac{1}{i}=(\frac{{K}_{M}^{app}}{{i}_{max}})(\frac{1}{C})+(\frac{1}{{i}_{max}})$$

where i is the current, i_max_ is the maximum current measured under saturated substrate conditions, and C is the glucose concentration. The results showed that ${{\rm{K}}}_{{\rm{M}}}^{{\rm{app}}}$ was 0.34 mM. A small ${{\rm{K}}}_{{\rm{M}}}^{{\rm{app}}}$ indicated that immobilised GOx possessed high enzymatic activity, and that the proposed electrode had high affinity for glucose. The sensitivity of the fabricated biosensor was higher than the previously reported ones, and its ${{\rm{K}}}_{{\rm{M}}}^{{\rm{app}}}$ was relatively low, as listed in Table 1.

**Table 1:** Comparison of different ZnO nanorod electrodes obtained in the present and previous works.

Electrode materials	Linearity	Response time (s)	Sensitivity (µA/mM·cm^2^)	Michaelis–Menten constant (K_m_)	References
Nafion/GOx/ZnO/FcC_11_SH/Au	0.05–1.00 mM	n/a	27.80	2.95	^14^
Aminopropyl)methyldiethoxysilane (APS)/GOx/ZnO/Si	0.02–1.60 mM	12	17.72	1.37	^37^
l-Cys /GOx/PVA/ZnONF/Au	0.25–19.00 mM	4	70.20	2.19	^61^
Nafion/GOx/ZnO/Zn	6.60 µm–0.38 mM	9	35.10	0.15	^32^
Nafion/GOx/AuNPs/ZnO/FTO	3.00 µm–3.00 mM	n/a	0.043	0.78	^41^
Nafion/GOx/ZnO/ITO	0.05–1.00 mM	10	48.75	0.34	This work
	1.00–20.00 mM		3.87		

### Interference studies, repeatability and reproducibility of the modified electrode

**Figure 8:**
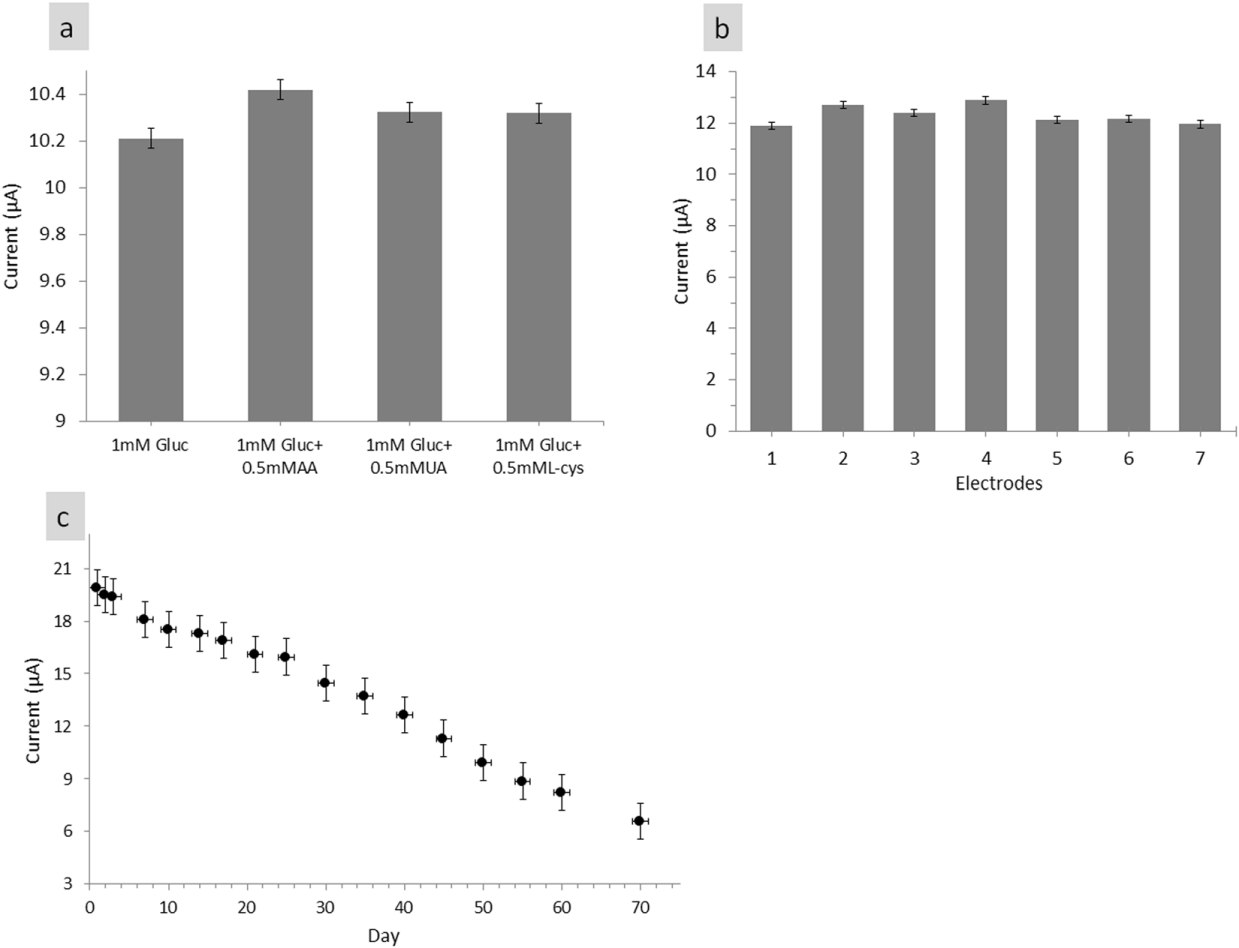
(**a**) Effect of interfering species of with 0.5 mM AA, UA and L-cysteine (L-cys) each added to 1 mM glucose on Nafion/GOx/ZnO NR-modified electrode, (**b**) Reproducibility of Nafion/GOx/ZnO NR-modified electrode current response on 7 different electrode on 3 mM of glucose solution and (**c**) The stability and lifetime of Nafion/GOx/ZnO NR electrode current response on 3 mM f glucose solution over 70 days.

Selectivity is one of the important characteristics of high-performance glucose biosensors. Two major factors that affect the selectivity of these sensors are enzyme–analyte reaction and selective measurement. According to Fu *et al*.^59^, the coelectro-oxidation of the potential interferences, such as AA and UA, affects the accuracy of glucose performance, although the content of interfering species to glucose was relatively low (3–8 mM) in physiological samples. Selectivity and interference studies were conducted by analysing a standard solution of 1 mM glucose with 0.5 mM interfering species. Figure 8(a) shows that with 0.5 mM AA, UA and L-cysteine (L-cys) added to 1 mM glucose, the current response slightly increased. As shown in Fig. 8(a), current response ranged from 2.03% (AA), 1.10% (UA) and 1.04% (L-cys) with respect to the current response to 1 mM glucose. Although a small increase in current response was caused by interfering species, these results can be excluded because the maximum concentration of these interfering species in the human body was approximately 0.1 mM^4^. Thus, the fabricated Nafion/GOx/ZnO NR electrode was selected for glucose and can be used for glucose determination in human serum samples under physiological conditions.

Repeatability refers to the agreement between successive measurements of the same sample, whereas reproducibility is defined as the closeness of agreement between results obtained using the same method under different conditions (different electrodes)^48^. The repeatability of glucose biosensor was calculated by analysing current response in 3 mM glucose for 20 times in a single day. As for reproducibility, current was measured in 3 mM glucose for seven different Nafion/GOx/ZnO NR electrodes (Fig. 8[b]). The 20 repeated measurements gave a relative standard deviation (RSD) of 2.99%, whereas the repeatability had an RSD of 3.03%. The RSD was calculated using the following equation^48^:

14 $$RSD\,( \% )=\frac{Standard\,deviation}{Average}\times 100 \% $$

The stability and lifetime of Nafion/GOx/ZnO NR electrode were evaluated by measuring the current response in 3 mM glucose for 70 days. The modified electrode was stored under dry conditions at 4 °C when not in use. As observed in Fig. 8(c), the modified electrode retained 90% of its initial response after 7 days, 85% after 14 days and 57% after 35 days. Zhao *et al*.^41^ fabricated ZnO NRs decorated with Au NPs on F-doped tin oxide electrode which retained a high stability of 87% of its initial current response after 14 days. Meanwhile, Zhou *et al*.^42^ reported a stability of 80.8% of its original current response in glucose after 10 days for ZnO NTs formed through the selective dissolution of ZnO NRs which were hydrothermally grown on Au cylindrical spiral electrode. This result was comparable with that of Zhao *et al*.^41^, who also indicated^42^ good affinity of GOx immobilisation onto ZnO nanorods surface even when simple physical adsorption was used.

### Reliability as biosensor in real sample

The reliability of fabricated Nafion/GOx/ZnO NR electrode in detecting glucose concentration in real blood samples was analysed. The blood samples were first analysed using Accu-Check Active (Model GB), and then amperometric analysis was performed by adding 5 µL of blood samples taken at different times (before meals, after breakfast and after lunch) and used without further pretreatment, except for diluting into 5 mL of PBS (0.01 M, pH of 7). The blood glucose concentration was calculated according to the linear equation of the fabricated Nafion/GOx/ZnO NR electrode. The results showed comparable performance with glucometer Accu-Check Active (Model GB), as shown in Table 2 (average values for three different electrodes). Nafion/GOx/ZnO NR electrode possessed good selectivity towards glucose detection against other interference species, such as AA and UA.

**Table 2:** Reliability of Nafion/GOx/ZnO NR electrode in real blood samples.

Samples	Determined by Accu-Check Active (Model GB)	Determined by Nafion/GOx/ZnO NR electrode
Before meals	4.3	4.02 ± 0.32
After breakfast	5.7	5.11 ± 0.90
After lunch	8.6	8.38 ± 0.29

## Conclusion

Highly oriented ZnO nanorods were formed on ITO-seeded substrates via hydrothermal method. The produced ZnO nanorods showed a good and suitable matrix for GOx immobilisation due to its good enzyme retention. DET between GOx and ZnO nanorods was achieved, resulting in good catalysis of GOx towards glucose. The relationship between the pH and temperature of electrolyte and GOx concentration used for immobilisation on the performance of the modified electrode was established. The produced Nafion/GOx/ZnO NR electrode exhibited a Michaelis–Menten constant of K_m_ = 0.34 mM which indicated that the immobilised enzyme had a strong affinity for glucose. The fabricated electrode also showed a high sensitivity of 48.75 µA/mM·cm^2^ within the linear range of 0.05 to 1 mM and good resistance towards interfering species. The modified electrode showed comparable results with a well-established glucometer to determine glucose in blood samples at different concentrations. Therefore, the produced Nafion/GOx/ZnO NR electrode is expected to have potential applications in real sample analysis.

### Experimental details

#### Synthesis and characterisation of ZnO nanorods/ITO electrode

ZnO NRs were grown on ITO substrates through a hydrothermal process assisted by ZnO seed layer growth. ITO substrates were cut and cleaned with NH_4_OH, H_2_O_2_ and distilled H_2_O at a ratio of 1:4:20 at 60 °C for 20 min and then rinsed with distilled H_2_O. ZnO seed solutions were prepared by dissolving 0.5 M ZnAc_2_·2H_2_O in methanol and stirred vigorously at 60 °C for 20 min. Then, ethanolamine was added dropwise into the solution as a stabiliser. The solution was stirred continuously at 60 °C for 2 h and aged at room temperature for 24 h before deposition. A ZnO seed layer was dropped onto the ITO substrate and dried at 150 °C for 20 min. This process was repeated thrice. After deposition, the coated ITO substrates were annealed at 500 °C for 2 h in air. Next, ZnO NRs were synthesised using a hydrothermal approach under optimum conditions previously reported by Ridhuan *et al*.^60^. ZnO NRs were initially grown in an aqueous solution containing 1:1 molar ratio of Zn(NO_3_)_2_ and HMT as a precursor solution. Then, hydrothermal process was performed in a preheated oven at 80 °C for 4 h. After reaction completion, the samples were rinsed with distilled water to remove any residual salt on the surface and dried in an oven at 90 °C for 1 h. The morphology and structure of the prepared ZnO seed layer and ZnO NRs were observed by FESEM (Zeiss Supra^TM^ 35VP, Carl Zeiss), whereas phase presence and crystallinity were analysed by XRD (Bruker AXS D8 Advance XRD).

### Glucose sensor electrode fabrication and electrochemical testing

**Figure 9:**
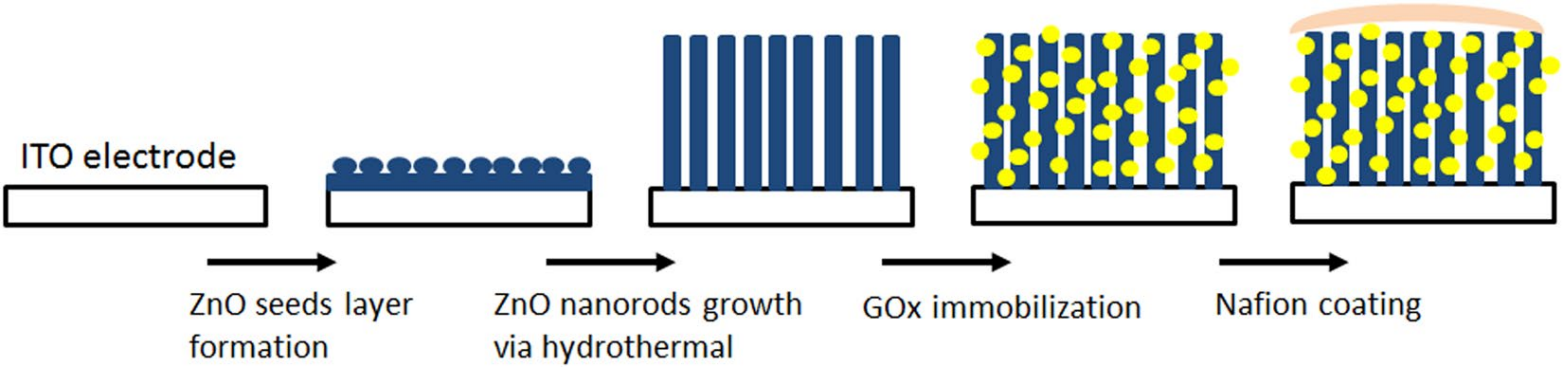
Schematic of ZnO nanorod array fabricated by hydrothermal method on ITO substrate. GOx and nafion were immobilised onto ZnO nanorods for glucose detection.

Prior to glucose biosensor fabrication, ZnO NRs were rinsed with PBS solution to generate a hydrophilic surface. To immobilise GOx onto ZnO nanorods, we dropped 10 μL of GOx solution onto the surface of ZnO NRs/ITO electrode and stored at 4 °C overnight, followed by extensive washing step to remove mobile GOx. Finally, 10 μL of 5% Nafion was dropped onto GOx/ZnO NRs/ITO. Figure 9 shows the schematic of ZnO nanorods array fabricated using hydrothermal method on an ITO substrate for glucose biosensor. Several variables were studied on the modified electrodes for glucose biosensor analysis. The optimum pH for enzyme activity was studied by varying the pH from 4 to 8; the temperature was varied from 10 °C to 60 °C. GOx concentration (1–10 mg/mL) immobilised onto the ZnO NR electrode was studied. Electrochemical measurement was performed using Portable Bipotentiostat/Galvanostat µSTAT 400 purchased from DropSens (Asturias, Spain) with a three-electrode system. All modified electrodes were stored at 4 °C under a dry condition until further usage. To demonstrate the practical usage of the modified electrode in real samples, we performed glucose detection in blood. Approximately 5 µL of human blood was collected at different times (before meals, after breakfast and after lunch) and used without further pretreatment except dilution in 5 mL of 0.01 M PBS (pH of 7) and stirred for 60 s. Glucose concentration level in blood samples was predetermined with Accu-Check Active (Model GB). Informed consent was obtained from subjects before collecting blood samples for glucose testing.
